# Reviewed article: Biase, TMMA; Silva, MT; Galvão, TF. Selective reporting of adverse renal reactions associated with selective cyclooxygenase-2 inhibitors in randomized clinical trials: a cross-sectional study, 2023. Epidemiol Serv Saude. 2026:35;e20260191

**DOI:** 10.1590/S2237-96222026v35e20260191.b

**Published:** 2026-11-09

**Authors:** Cristiane Menezes de Padua

**Affiliations:** 1 Universidade Federal de Minas Gerais, Belo Horizonte, MG, Brazil Universidade Federal de Minas Gerais Belo Horizonte MG Brazil

## Peer review B

### Questionnaire

Does the manuscript contain new and significant information to justify publication? Yes 

Does the Abstract (Summary) clearly and accurately describe the content of the article? Yes 

Is the problem significant and concisely stated? Yes 

Are the methods described comprehensively? Yes 

Are the interpretations and conclusions justified by the results? Yes 

Is adequate reference made to other work in the field? Yes 

### Manuscript Structure:

Length of article is: Adequate

Number of tables is: Adequate

Number of figures is: Adequate

### Rating

Interest Good

Quality Good

Originality Good

Overall Good

Recommendation: **Minor Revision**

## Comments

Comentários gerais

O artigo apresenta uma análise do relato seletivo de reações adversas renais aos inibidores seletivos da ciclo-oxigenase-2 em ensaios clínicos a partir de uma revisão sistemática publicada em 2024. O tema é relevante e contribui para a discussão sobre aspectos metodológicos e éticos na condução de ensaios clínicos relativos à segurança do uso de medicamentos. Outro aspecto importante é a disponibilidade dos dados, o que confere transparência ao manuscrito.

Comentários específicos:

- Rever “tradicionais” na frase “... em comparação aos anti-inflamatórios não esteroides tradicionais” - “disponíveis” seria uma opção (pág. 2).
- No trecho “Logo após seu uso generalizado, surgiram preocupações quanto à segurança cardiovascular [2], levando à retirada do mercado do rofecoxibe em 2004, o primeiro coxib a ser descontinuado por esse motivo [3, 4]”, recomendo fortemente que os autores detalhem esse processo de retirada do medicamento, ilustrando, inclusive, que no relato dos resultados do estudo VIGOR houve omissão de eventos adversos importantes, incluindo os cardiovasculares (ver referência https://www.nejm.org/doi/full/10.1056/ NEJMe058314) (pág. 2).
- No trecho “A omissão de reações adversas a medicamentos nas publicações ... a riscos desnecessários [9]”, avaliar a substituição de riscos desnecessários por “danos graves” ou equivalente que traduza melhor a gravidade dos efeitos adversos renais (pág. 2).
- Sugiro apresentar o conceito de relato seletivo adotado na introdução junto ao trecho “O relato seletivo desses eventos adversos foi frequente, contribuindo para o risco de viés e imprecisão dos resultados.” (pág. 2)
- Explicitar como a variável dependente (relato seletivo) foi dicotomizada no início da seção “Métodos”.
- Com relação às variáveis, minha principal ressalva é a definição do tempo de revisão por pares e seu uso como uma proxy de qualidade de revisão por pares. Primeiramente, o tempo decorrido entre a data de submissão e aceite pode depender de outros fatores não “controláveis” dentro do processo de avaliação do artigo, cuja informação não pode ser acessada pelos autores. Acredito que o termo mais apropriado seria tempo de processamento editorial, como os próprios autores denominaram na página 4 (4° parágrafo, seção resultados). Sugiro que os autores incluam essa questão nas limitações do trabalho, apreciando ainda sua utilização como uma variável independente, sugestiva de qualidade da revisão por pares.
- Acredito que uma descrição detalhada dos relatos seletivos (variável desfecho) na seção Resultados agregaria valor ao artigo. Sugiro apresentar esta descrição em uma tabela/material suplementar.
- Avaliar mover o 2° parágrafo da discussão (Esta análise baseou-se... interpretação dos achados [14, 15]) para os parágrafos finais artigo, contemplando a limitação da variável tempo de revisão por pares.
- Resumo: rever a escrita dos objetivos “Identificar frequência e fatores associados” (ex. calcular frequência e identificar fatores associados...)

